# The Correlation between Binding and Transport of a Siderophore Complex through Its TonB-Dependent Transporter

**DOI:** 10.1021/acsomega.5c10450

**Published:** 2026-03-02

**Authors:** Matteo Ceccarelli, Aravind Selvaram Thirunavukarasu, Stefan Milenkovic

**Affiliations:** Department of Physics, University of Cagliari, Cittadella Universitaria, Monserrato 09042, Italy

## Abstract

A promising approach to deliver antibiotics into Gram-negative bacteria is the “Trojan Horse” strategy, which exploits bacterial iron uptake systems to facilitate transport across the outer membrane. Only one “Trojan Horse” antibiotic has reached the market, underscoring the need to deepen our understanding of this mechanism. In this study, we investigate the molecular mechanisms underlying siderophore-mediated transport through PfeA, a TonB-dependent transporter from *Pseudomonas aeruginosa*, using a variety of modeling techniques, and analyze modalities of substrate translocation across the outer membrane. We reconstruct the passage of the Fe^3+^–enterobactin complex through PfeA, revealing distinct roles for the first and second binding sites. The first binding site initiates signal propagation toward the TonB box, while the deeper second binding site facilitates progressive ligand migration by weakening plug–barrel interactions. Thus, destabilization of plug–barrel hydrogen bonds—rather than movement of the plug domain—triggers substrate translocation. These findings provide mechanistic insight into the molecular basis of siderophore uptake and help clarify how specific binding and conformational events can facilitate substrate translocation through TonB-dependent transporters.

## Graphical abstract

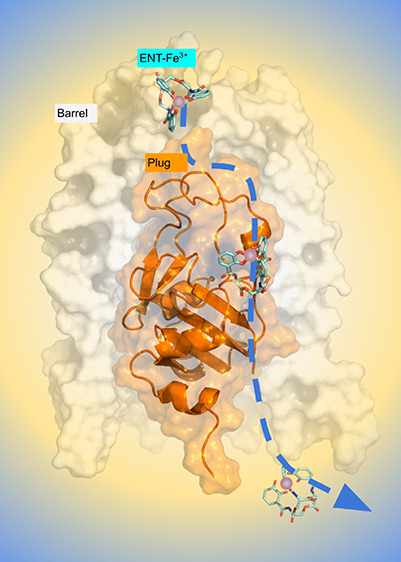

## 1 Introduction

Since the 1980s,^1,2^ the iron uptake pathway in Gram negative bacteria has been explored by several research groups and pharmaceutical companies aiming to develop “Trojan Horse” compounds with enhanced permeability.^3–5^ This approach involves designing antibiotics that can bind iron and thus would exploit siderophore-dependent uptake systems to cross the tight outer membrane.^6^ The simplest implementation involves creating a molecular scaffold by conjugating an antibiotic with one or more high-affinity iron-chelating groups, such as catechols or hydroxamates.^7^ Despite the conceptual and chemical simplicity of this strategy, only one siderophore-conjugated antibiotic, cefiderocol,^8^ has been approved, with another, GT-1, currently in Phase 3 clinical trials.^9^ These low numbers likely reflect multiple factors, including toxicity, limited antibacterial spectrum, and variable in vivo efficacy, while also highlighting remaining knowledge gaps and the need to deepen our fundamental understanding of how molecules traverse the outer membrane by hijacking siderophore transporters.^3^ Interestingly, in some cases, these transporters can also be triggered by molecules that do not bind iron,^10,11^ further underscoring the need to expand our basic knowledge of these mechanisms. Siderophore transporters belong to TonB-dependent transporters (TBDT) sharing a common beta-barrel architecture with porins, which involves 22 antiparallel β-sheets that facilitates a plug (cork) domain in its interior.^12,13^ The longer extracellular loops, together with the “outer” portion of the plug domain create ligand-binding site(s), and for this reason are also referred as ligand gated pores (LGP).^14^ Upon specific binding,^15^ a signal to the N-terminus of the plug domain, the TonB-box, promotes its interaction with TonB. Recent AFM investigations suggested that this noncovalent interaction is sufficiently strong to allow the pulling and partial unfolding of the entire plug domain via the proton motive force system (PMF), creating a pore large enough for the translocation of molecules before the detachment of TonB.^16^ Thus, the mechanism can be depicted in two steps, the uptake, namely binding and activation of the transporter, followed by the transport, namely opening of transporter and internal translocation. However, because accommodating the complete unfolded plug, as suggested in ref 16, demands considerable space (>20 nm) and energy (50 pN × 20 nm), a more precise model formulation is required.

In this context, PfeA was selected as a representative TonB-dependent transporter because of its central role in iron uptake in *Pseudomonas aeruginosa*, a pathogen in which molecular transport across the outer membrane is known to be largely independent of general porins. Recent experimental evidence has shown that small-molecule accumulation in *P. aeruginosa* relies predominantly on specific uptake pathways rather than nonspecific porin-mediated diffusion, highlighting the importance of dedicated transporters for antibiotic entry.^17^ PfeA is among the best-characterized siderophore receptors in this organism, with available high-resolution structural data and a well-established two-site binding model. These features make PfeA a biologically relevant and mechanistically informative system for investigating siderophore-mediated transport across the outer membrane.

With the aim to assist the development of new siderophore compounds, we focus our attention on the opening of the pore and the internal translocation by which molecules reach the periplasm via diffusion. Starting from our assessed two-site model as a trigger for the uptake mechanism of enterobactin+iron complex (Fe^3+^–ENT) in PfeA,^18^ by combining standard MD, docking, and metadynamics simulations we have managed to reconstruct the complete passage of the iron complex through the transporter, suggesting a new mechanism with its molecular details.

## 2 Methods

### 2.1 Molecular Dynamics Simulations

The systems were prepared following ref 18. The PfeA (PDB ID: 6Q5E) protein was embedded in a phospholipid bilayer and fully solvated using the CHARMM-GUI web server.^19^ The bilayer consisted of 233 POPC (1-palmitoyl-2-oleoyl-*sn*-glycero-3-phosphocholine) molecules with in-plane dimensions of 100 × 100 Å. The system was immersed in explicit water and neutralized with KCl at a concentration of 0.15 M.

Molecular dynamics (MD) trajectories were generated using ACEMD.^20^ The AMBER14 force field was applied to the protein and LIPID14 to the lipids,^21,22^ while water molecules were modeled using the TIP3P model.^23^ Parameters for the Fe^3+^–enterobactin complex were derived using the Metal Center Parameter Builder (MCPB).^24^

### 2.2 Cavity Analysis

Cavities were identified using VOIDOO^25,26^ with a probe radius of 1.5 Å, corresponding approximately to the size of a water molecule, and an initial grid spacing of 1.0 Å. The grid was refined iteratively until the cavity volume converged within 0.1% between successive evaluations. Cavity data from all simulations were subsequently subjected to cluster analysis using a 3.0 Å RMSD cutoff.

### 2.3 Correlation Analysis of Protein Motions

C_α_ correlation matrices were computed using the bio3d package in R^27^ employing the linear mutual information (LMI) approach.^28^ Compared to traditional correlation measures, LMI is invariant to the orientation of atomic fluctuations and thus provides a more detailed and physically meaningful description of correlated motions. Convergence was assessed by dividing the full trajectory into 50 ns blocks and calculating the correlation matrix for each window.

### 2.4 Molecular Docking

Docking calculations were performed using AutoDock Vina.^29^ The search space was defined as a rectangular box of 60 × 60 × 60 Å^3^ centered at (0.0, 0.0, 20.0) Å, fully encompassing the protein. The exhaustiveness parameter was set to 4096 (default value = 8). Enterobactin coordinates were kept fixed at the X-ray geometry, while PfeA configurations were taken from both the X-ray structure and selected snapshots from MD simulations.

In order to improve the statistical robustness of the docking analysis and to partially account for protein conformational variability, docking calculations were performed on an ensemble of approximately 1000 PfeA structures extracted from equilibrium MD simulations. Each snapshot was treated as a rigid receptor during the docking procedure.

This ensemble-based docking strategy was adopted to avoid reliance on a single protein conformation and to ensure that the identified docking regions reflect recurrent geometric features of the transporter rather than artifacts of a specific snapshot. Docking was therefore used as a structural filtering step to identify regions of PfeA that are consistently compatible with the Fe^3+^–enterobactin complex across the MD ensemble.

### 2.5 Metadynamics Simulations

Metadynamics simulations were performed by combining ACEMD with PLUMED2.^30^ The collective variables employed were: (i) the distance between the centers of mass of the Fe^3+^–enterobactin complex and PfeA projected along the *z*-axis, and (ii) the number of contacts between the ligand and the protein. In addition, a predefined external bias was applied with respect to (iii) the number of plug–barrel hydrogen bonds and (iv) the position of the plug center of mass relative to the barrel center of mass. In order to be faster and efficient in reconstructing the underlying free energy, we employed several replica of the system exploring the same reduced space of the defined collective variables. Each replica works as an independent walker subjected to a common bias contributed by all, thus the free energy is reconstructed by multiple walkers.^31^

The metadynamics-to-dynamics approach^32^ was used to calculate the residence time of the ligand within the protein. A total of 24 simulations were performed, comprising 12 simulations for the wild-type system and 12 for the double mutant. Each simulation was stopped when the Fe^3+^–enterobactin complex detached from the proposed X-ray binding site. The resulting residence times were fitted to the corresponding probability density function, and the Kolmogorov–Smirnov test^33^ was employed to assess the quality of the fit.

## 3 Results

### 3.1 Residence Time Analysis

We began by evaluating the first binding site (FBS) residence time distribution of the Fe^3+^–ENT in wild-type (WT) PfeA and in the double mutant R480A/Q482A. The R480A/Q482A double mutant was selected because residues R480 and Q482 are located in the extracellular loop region of PfeA that forms part of the primary binding site for ferric enterobactin. Previous structural and functional studies have shown that mutation of these residues strongly impairs siderophore binding and transport, making this variant a well-established transport-deficient control for probing the uptake mechanism.^18,34^ Residence times were obtained by using the metadynamics-to-dynamics algorithm.^32^ As shown in Figure 1, the residence time in the WT system is approximately 4 orders of magnitude longer than in the mutant. Moreover, in the WT, there exists a small but finite probability for the complex to progress toward the second binding site (SBS), whereas this progression is completely absent in the mutant (Figure S1). These findings support the conclusion that a sufficiently long residence time which reflects high-affinity binding is a necessary condition for successful transport of the substrate.

**Figure 1:**
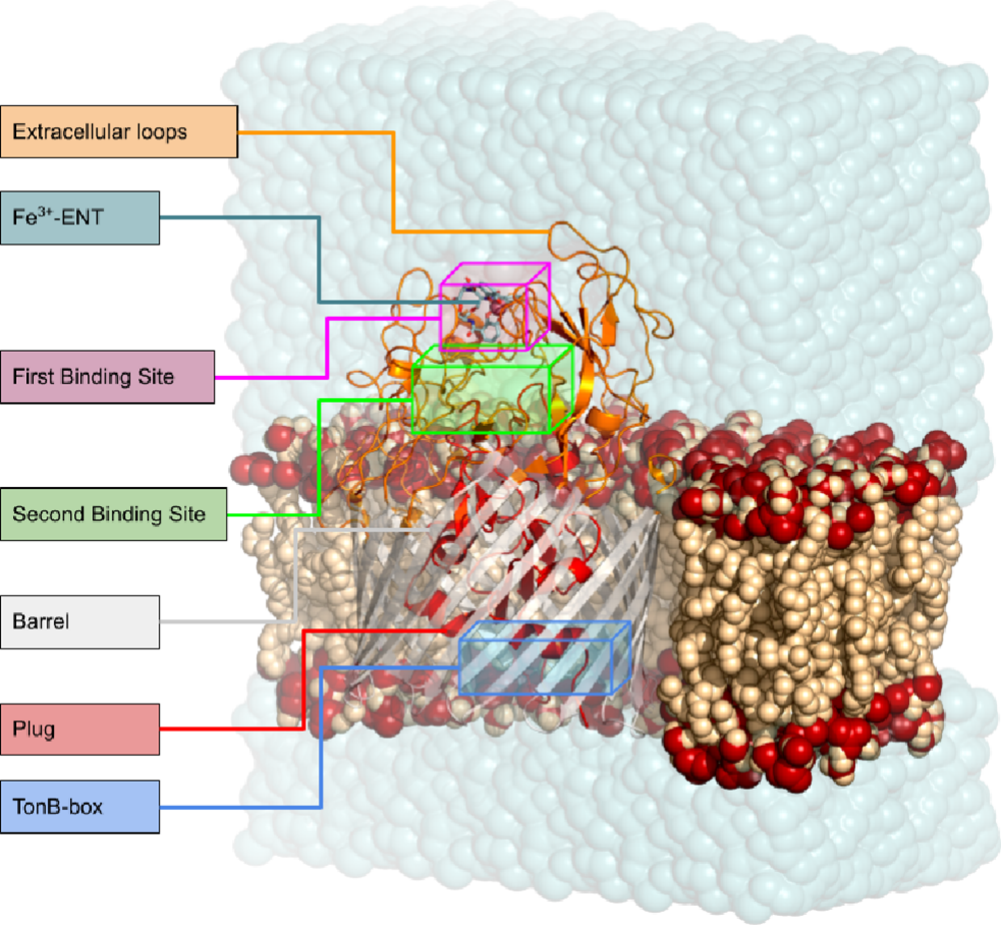
Structural overview of the PfeA transporter. Schematic representation of the TonB-dependent transporter PfeA embedded in the outer membrane. The β-barrel, internal plug domain, TonB box at the N-terminus, and extracellular loops are indicated. The locations of the first (FBS) and second (SBS) binding sites for the Fe^3+^–enterobactin complex are highlighted, together with the relative orientation of the extracellular and periplasmic sides of the membrane. This figure provides a reference framework for the transport mechanism discussed throughout the manuscript.

### 3.2 Volume Analysis

To better understand the interaction between PfeA and the Fe^3+^–ENT complex, we investigated where the ligand could be accommodated within PfeA. Beyond its role in signaling, ligand binding is believed to induce conformational changes in the plug domain relative to the β-barrel, facilitating the transport of structurally diverse substrates.^35,36^ Previous studies have shown that the space between the plug and barrel is filled with water—up to ∼ 100 molecules—and stabilized by approximately 40 hydrogen bonds.^37,38^ This suggests that the plug domain retains a degree of mobility within the barrel. Based on this, we hypothesized that ligand binding might trigger the formation of a lateral pore, sufficiently large to permit siderophore diffusion.

To test this, we analyzed the internal cavity distribution of PfeA in both the apo and Fe^3+^–ENT-bound states. In the apo form, we identified 18 distinct cavities, while in the bound form this number decreased to 14. However, as shown in Figure S2, the remaining cavities in the Fe^3+^–ENT-bound complex exhibit significantly larger average volumes—particularly those exceeding 500 Å^3^, which are capable of accommodating the ligand. Notably, one such enlarged cavity is located in a lateral corridor between the plug and β-barrel domains, centered along the *Z*-axis. This suggests that Fe^3+^–ENT binding induces structural reorganization that enlarges specific hollow regions, potentially facilitating internal migration of the complex.

### 3.3 Docking

While cavity size is a necessary condition for ligand accommodation, it is not sufficient on its own. To identify which internal voids best match the physicochemical properties of the Fe^3+^–ENT complex, we conducted an extensive docking campaign. This involved ∼1000 representative conformations extracted from our PfeA– Fe^3+^–ENT standard MD simulations.

Beyond the immediate goal of identifying structurally compatible docking regions, this extensive statistical sampling represents a deliberate investment toward future data-driven analyses. Multiple studies have demonstrated that statistical descriptors extracted from molecular dynamics ensembles can encode rich information about molecular recognition and transport phenomena, enabling the prediction of thermodynamic and functional properties using machine learning approaches.^39–41^ In this context, the use of a large ensemble of MD-derived receptor conformations allows docking results to be interpreted not as isolated poses, but as statistically meaningful patterns that can be integrated with cavity analysis and enhanced-sampling simulations.

A summary of the docking and cavity volume analysis is presented in Figures 2 and S3. Multiple favorable docking sites were identified, with the most energetically favorable and frequently sampled pose located within the previously described lateral corridor—which we designate as the internal docking site (IDS).

**Figure 2:**
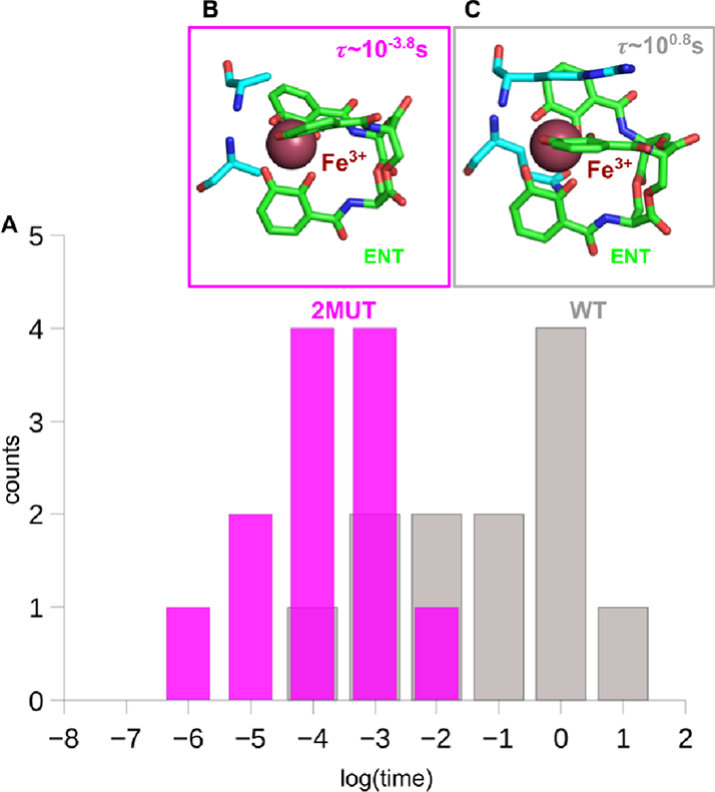
Residence time distributions and ligand coordination in WT and mutant systems. (A) Distributions of residence times obtained from metadynamics-to-dynamics simulations for the wild-type (WT, gray) and double mutant (2MUT, magenta) PfeA systems. (B) Representative coordination geometry of the Fe^3+^–enterobactin complex in the 2MUT system. (C) Representative coordination geometry of the Fe^3+^–enterobactin complex in the WT system. Insets in panels B and C illustrate the different local interaction patterns between the ligand and the transporter in the two systems.

**Figure 3:**
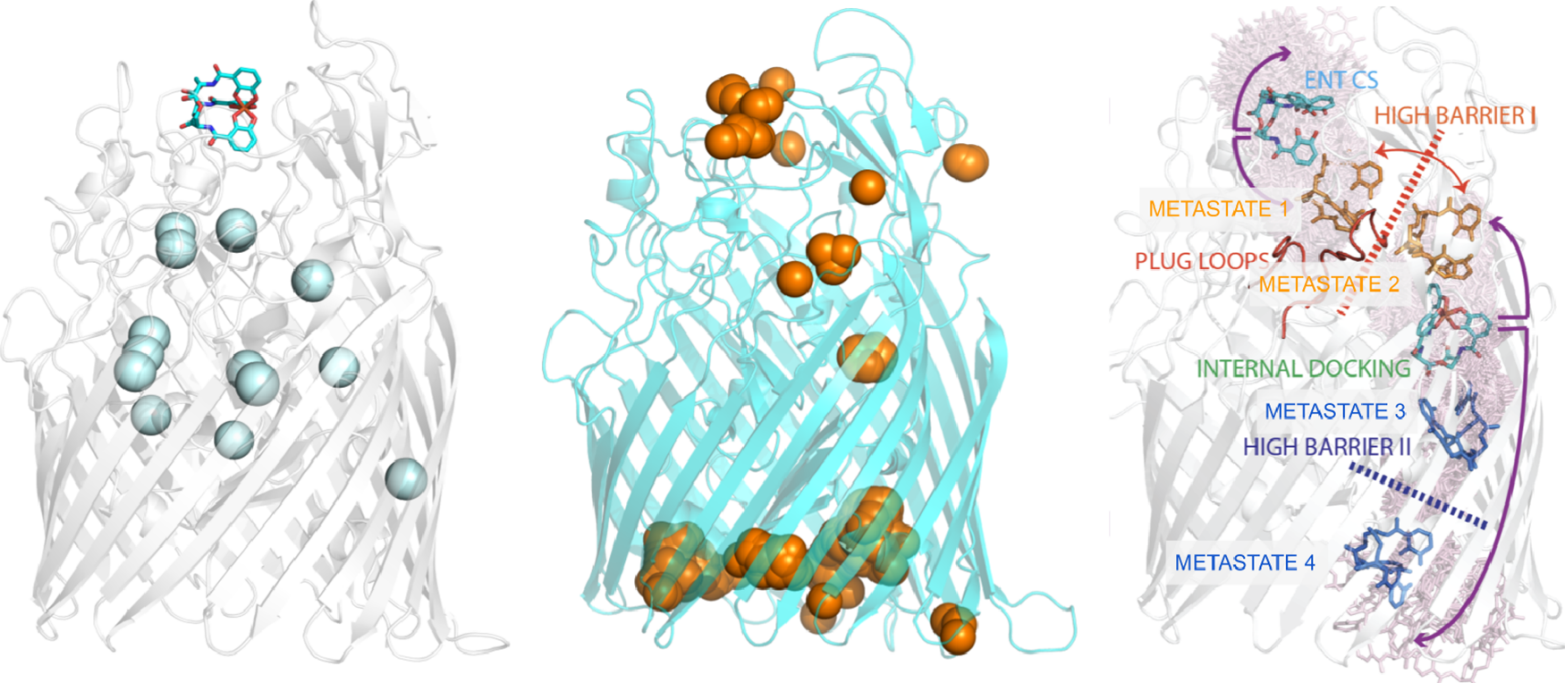
Comparison of the results of the three independent methods applied to PfeA. Left: results of cavity calculations, the cyan spheres being the position of internal voids. Enterobactin is shown in the position extracted from crystal structure. Center: results of docking campaign for enterobactin on PfeA, the orange spheres being the poses. Right: superimposition of sample positions of enterobactin obtained with multiple walkers metadynamics simulations starting from (i) the crystal structure position (FBS), (ii) the SBS, and (iii) IDS. Two high free energy barriers are identified.

**Figure 4:**
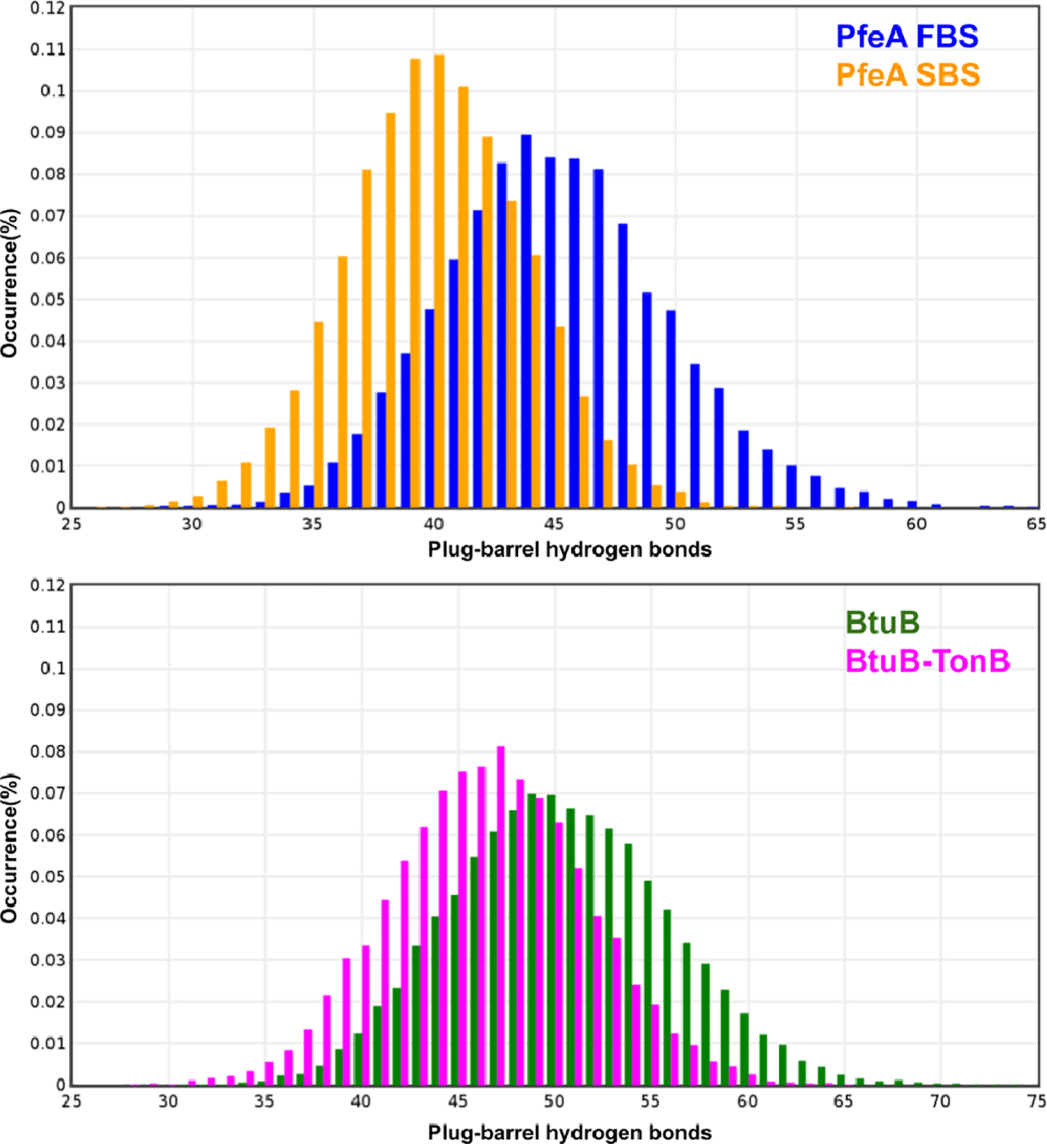
Plug-barrel interactions. Distributions of plug-barrel hydrogen bonds during standard MD simulations, on top for the PfeA siderophore transporter with enterobactin bound in the two sites, on bottom for the BtuB transporter, without and with TonB bound.

Interestingly, docking to the SBS was less prominent than anticipated. This may be due to conformational rearrangements observed in prior MD simulations,^18^ which reinforce the idea that a prolonged residence time at the FBS is essential for successful progression toward the SBS. This observation is consistent with a two-step mechanism previously proposed for the translocation of a substantially larger and more complex substrate, colicin B, through FepA, a PfeA homologue in *E. coli*.^42^

### 3.4 Multiple-Walkers Metadynamics

With three plausible binding positions for Fe^3+^–ENT—the FBS, the SBS, and IDS—we next sought to assess their functional relevance. To this end, we first performed standard MD simulations with the ligand positioned in either the SBS or the IDS. Both systems exhibited structural stability, as confirmed by converged RMSD profiles (Figure S4).

Interestingly, when Fe^3+^–ENT resides in the SBS, long-range correlations toward the TonB-box are not maintained (Figure S5A). Instead, we observe strong correlations between plug-domain loops, suggesting that ligand binding in the SBS may promote local conformational changes within the plug, necessary to initiate the next step of the transport process.

We then performed multiple-walkers metadynamics (MTD) simulations,^43^ distributing the walkers across the three binding sites (FBS, SBS, IDS) as well as near the periplasmic exit of PfeA. The goal of this approach was to reconstruct a free energy profile for Fe^3+^–ENT translocation under the condition that the plug domain remains fully intact—i.e., without unfolding. As collective variables (CVs), we selected: (i) the *z*-component of the center-of-mass (COM) distance between Fe^3+^–ENT and the protein, and (ii) the number of contacts between them (Figure 5).

**Figure 5:**
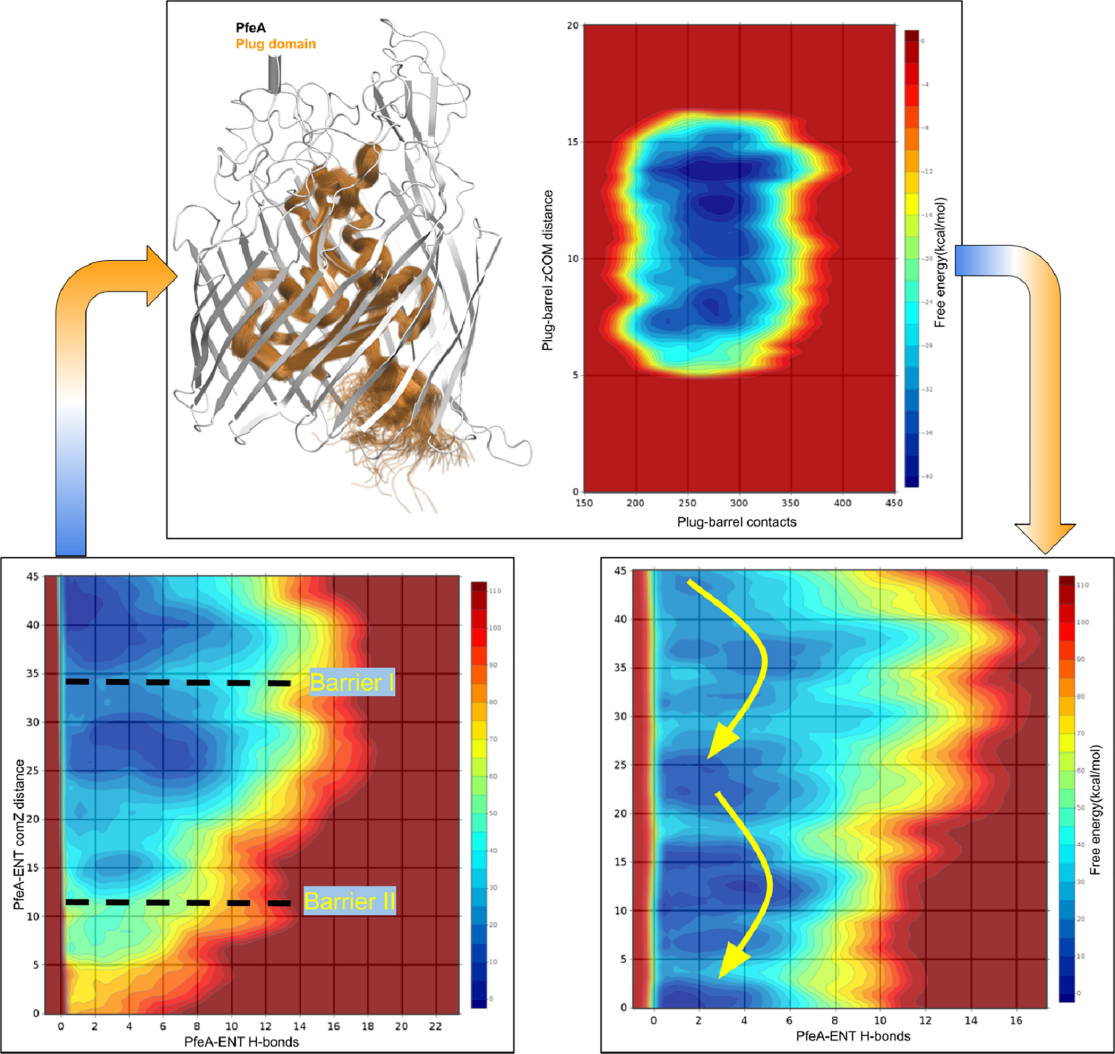
BOTTOM: Reconstructed free energy surfaces from multiple walkers metadynamics of enterobactin diffusing inside PfeA without (left) and with a bias (right). TOP: effect of the applied bias to the plug (left), and quantification of plug-barrel bias used within multiple walkers metadynamics (right).

Despite applying bias potentials, we did not observe a continuous sampling trajectory from the FBS to the periplasm. As shown in Figure 2, where we superimposed the different sampled conformations of Fe^3+^–ENT inside PfeA from metadynamics, we revealed the presence of two distinct high-energy barriers along the path. These discontinuities in sampling reflect strong interactions between the plug and barrel domains that hinder translocation. The first barrier arises during the transition over the plug loops, from the SBS to the lateral corridor toward the IDS. The second occurs at the exit from the IDS into the periplasmic space, the region near the TonB-box.

### 3.5 Plug–Barrel Hydrogen Bonds

To better understand the origin of the transport barriers observed in our metadynamics simulations, we revisited the plain MD simulation of Fe^3+^–ENT bound in the SBS. Unlike the FBS-bound configuration, this simulation did not show strong correlation with the TonB-box region (Figure S5A). However, closer inspection revealed that binding of Fe^3+^–ENT at the SBS weakens the interaction between the plug domain and the β-barrel, as evidenced by a measurable reduction in the number of hydrogen bonds between them, as shown from the distributions in Figure 3. The hydrogen bonds disrupted at the plug–barrel interface appear to be partially compensated on the barrel side by increased interactions with water and by the formation of additional internal hydrogen bonds within the barrel. Conversely, the plug shows an increase in intradomain interactions, suggesting a redistribution of its internal hydrogen-bonding population (Figure S6).

This observation highlights the sensitivity of the plug–barrel hydrogen-bonding network to ligand binding, even for a relatively small substrate like Fe^3+^–ENT. It led us to ask: how would this network respond to a larger binding partner, such as TonB? To explore this, and in the absence of structural data for the PfeA–TonB complex, we conducted MD simulations of the homologous BtuB transporter both with and without TonB bound (PDB ID: 2GSK).^44^ As shown in Figure 3, the presence of TonB leads to a ∼20% decrease in plug–barrel hydrogen bonds compared to BtuB alone. Although we could not reconstruct the exact sequence of events Figure S7 shows that upon TonB binding effective area occupied by plug is decreased. This narrative is further supported by a decrease of plug-water hydrogen bonds and increase of barrel-water hydrogen bonds reported in panels C and D of Figure S7, respectively.

These results demonstrate that plug–barrel destabilization can occur spontaneously upon binding of either ligands or protein partners—and potentially through a cumulative effect. This raises a critical mechanistic question: if hydrogen bond disruption alone is sufficient to weaken the plug–barrel interaction, could this enable Fe^3+^–ENT translocation without requiring full or partial unfolding of the plug? And if so, is this pathway energetically more favorable than previously proposed mechanisms based on plug displacement or unfolding? ^16,36^

To test this hypothesis, we designed a dedicated MTD simulation in which the collective variables (CVs) were limited to (i) the number of hydrogen bonds between the plug and barrel domains, and (ii) the *z*-component of the center-of-mass (COM) distance between the plug and the rest of the protein. This approach enabled targeted exploration of how destabilization of the plug–barrel interface affects plug mobility. Remarkably, over the course of this simulation, we observed spontaneous initiation of plug unfolding. This result suggests that partial unfolding is energetically more favorable than displacing the plug as a rigid body—a conclusion consistent with previous findings.^36^

### 3.6 Repeated Metadynamics

We next used the free energy profile obtained from the plug–barrel-focused metadynamics as a biasing grid and repeated the MTD simulations, this time actively pulling the Fe^3+^–ENT complex across the membrane. This allowed us to assess: (i) how the previously observed free energy barriers would be affected, and (ii) which of the two collective variables—plug–barrel hydrogen bond count or plug displacement, would be preferentially sampled during transport.

As shown in Figure 4, the results reveal a striking change in the free energy landscape. The barrier below the internal docking site (IDS) is fully eliminated, allowing Fe^3+^–ENT to exit PfeA with minimal resistance. The barrier between the SBS and IDS is also significantly reduced—by approximately 8 kcal/mol—highlighting the substantial impact of the modified grid on facilitating transport. We report in Movie S1 the diffusion of the ligand from FBS to periplasmic space generated by extracting successive conformations from one of the MTD walkers.

To better understand the underlying mechanism, we analyzed the sampling behavior of the two grid variables. While disruption of plug–barrel hydrogen bonds was prominently explored, we also observed that, over the course of the extended simulation, spontaneous unfolding of the plug’s N-terminal region occurred concurrently. This unfolding—evident in the conformational changes shown in Figure S8—suggests that hydrogen bond destabilization and partial unfolding are not mutually exclusive, but may act cooperatively to reduce the energetic barriers to translocation.

## 4 Discussion

The transport model proposed in this study extends our previous findings^18,34^ and offers key advantages over previously suggested mechanisms, particularly in terms of energetic feasibility.^16,36^ Rather than requiring full displacement of the plug domain from the barrel through unfolding, which is a process that would demand breaking a large number of hydrogen bonds and create spatial constraints incompatible with the periplasmic environment, our simulations suggest that local disruption of the plug–barrel hydrogen bond network is sufficient to facilitate ligand translocation.

This is supported by our observation that binding of the relatively small Fe^3+^–ENT complex to the SBS induces a spontaneous reduction in plug–barrel hydrogen bonds (∼10%). When further explored via metadynamics simulations, this disruption can reach ∼40%, effectively lowering the free energy barrier for transport. Importantly, this bond weakening is accompanied by spontaneous, localized unfolding of the plug’s N-terminal region, suggesting that the two processes are cooperative rather than mutually exclusive. This interpretation is further supported by the observation that ligand-bound states in the SBS and IDS remain structurally stable on the time scale of standard MD simulations (Figure S4), while exhibiting distinct long-range correlation patterns within the plug domain (Figure S5). In particular, SBS binding disrupts correlations toward the TonB box while enhancing internal plug-domain coupling, consistent with a locally primed but not yet translocation-competent state.

Notably, hydrogen bonds stabilizing secondary structure are mostly backbone-mediated and geometrically constrained, rendering them energetically more robust, whereas hydrogen bonds stabilizing quaternary interactions are largely side-chain mediated and more exposed to solvent and local conformational fluctuations. This distinction, predicted by general theoretical models of protein stability and observed in hydrogen-bonding analyses across different protein systems, may facilitate selective disruption of interdomain contacts without compromising the overall fold.^45–48^ These findings align with previous single-molecule studies suggesting that mechanical remodeling, rather than complete plug unfolding, underlies TonB-dependent transport,^16^ providing energy through the proton motive force (PMF) to destabilize further the plug–barrel interactions.

While TonB-dependent transport is intrinsically a nonequilibrium process driven by continuous energy input from the proton motive force, the free-energy landscapes obtained here should be interpreted as effective, quasi-equilibrium descriptions of the dominant energetic bottlenecks. In this framework, ligand binding and TonB engagement reshape the underlying free-energy surface by modulating plug–barrel interactions, thereby lowering transport barriers that are subsequently crossed under nonequilibrium conditions (Figure 6).

**Figure 6:**
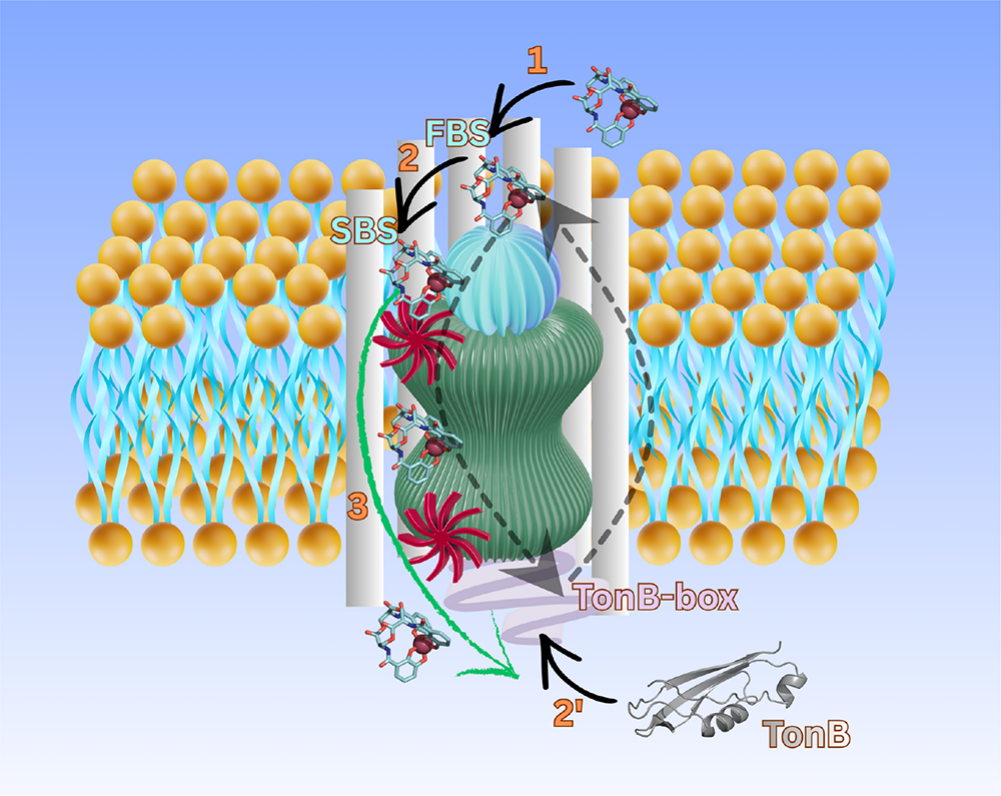
Proposed concerted mechanism for Fe^3+^–enterobactin transport through a TonB-dependent transporter. A possible cooperative action of Fe^3+^–enterobactin binding at successive sites and TonB engagement is illustrated. The previously observed correlation between ligand binding at the first binding site (FBS) and the TonB box is indicated by dashed arrows.^18,34^ High-energy barriers along the transport pathway are represented by turbine symbols.

To investigate whether such destabilization might also occur upon TonB binding, we performed comparative simulations of the BtuB transporter with and without TonB. In agreement with our hypothesis, the BtuB–TonB complex exhibited a ∼20% reduction in plug–barrel hydrogen bonds compared to the unbound state (Figure 3). This indicates that TonB binding alone can weaken the plug–barrel interface, and when combined with ligand-induced effects, may create a synergistic mechanism that primes the transporter for substrate translocation—ultimately completed by the proton motive force (PMF) system. Importantly, the energy associated with ligand binding and TonB engagement is not directly transmitted as a mechanical force acting on the substrate. Instead, this energy is transduced through progressive destabilization of the plug–barrel hydrogen bond network, which reshapes the free-energy landscape and lowers the barrier separating internal binding regions from the periplasm. Once this barrier is sufficiently reduced, substrate passage proceeds predominantly by diffusion. In this view, energy transduction is indirect and operates through modulation of protein–protein interactions rather than through active pulling of the ligand.

Further support for this model comes from our cavity analysis. Upon Fe^3+^–ENT binding, the total number of internal cavities within PfeA decreases, yet those remaining—particularly in the lateral corridor between the plug and barrel domains—exhibit increased volume, in agreement with the possibility to transport large substrates.^42,49^ This structural reorganization reflects an adaptive architectural response that enables the ligand to exploit internal pathways, such as the internal docking site (IDS), during translocation. The IDS should therefore not be interpreted as a stable or obligatory intermediate along the transport pathway. Rather, it represents a transient, structurally permissive region that is consistently identified by independent analyses. While ligand probability accumulates at the IDS, a substantial free-energy barrier separates this region from the periplasm. Only upon weakening of the plug–barrel hydrogen bond network does this barrier collapse, enabling ligand progression toward translocation.

## 5 Conclusions

Our computational study proposes an energetically viable mechanism for Fe^3+^–ENT transport through the TonB-dependent transporter PfeA in Gram-negative bacteria. We show that ligand progression from the first to the second binding site weakens the hydrogen bond network between the plug and barrel domains. Separately, simulations of the homologous BtuB–TonB complex reveal that TonB binding alone also reduces plug–barrel hydrogen bonding. These findings suggest that ligand binding and TonB interaction could potentially act in concert to facilitate local plug rearrangement, without requiring full plug unfolding—a process likely incompatible with the physical and energetic constraints of the periplasm. This mechanism refines our understanding of TonB-dependent transport and highlights a more nuanced model underlying the Trojan Horse strategy. By demonstrating how targeted disruption of internal hydrogen bonds can facilitate substrate passage, this work provides mechanistic insight into the factors that modulate transport through TonB-dependent transporters and establishes a conceptual basis for future studies aimed at understanding and potentially hijacking transporter activity.

## Supplementary Material





## Notes

Supplementary figures providing additional details for the results discussed in the main text; owing to the size of the primary trajectory data, further data sets can be obtained from the authors upon request (PDF)
Movie showing the diffusion of the Fe^3+^–enterobactin complex in the simulated system (MP4)
