# Supplementary material for: The Correlation between Binding and Transport of a Siderophore Complex through Its TonB-Dependent Transporter
Source: ACS Omega. 2026 Mar 2;11(21):30494–502. doi: 10.1021/acsomega.5c10450 (PMC13234889; doi:10.1021/acsomega.5c10450)
Supplement: Supplementary file 1 [file ao5c10450_si_001.pdf]

SUPPORTING INFORMATION

The correlation between binding and transport of a siderophore complex through its TonB-dependent transporter

Matteo Ceccarelli[1], Aravind Selvaram Thirunavukarasu[1], Stefan Milenkovic[1,*]

[1]*Department of Physics, University of Cagliari, Cittadella Universitaria, Monserrato, 09042 Italy*

*Corresponding author: smilenkovic@dsf.unica.it

Supporting Figures

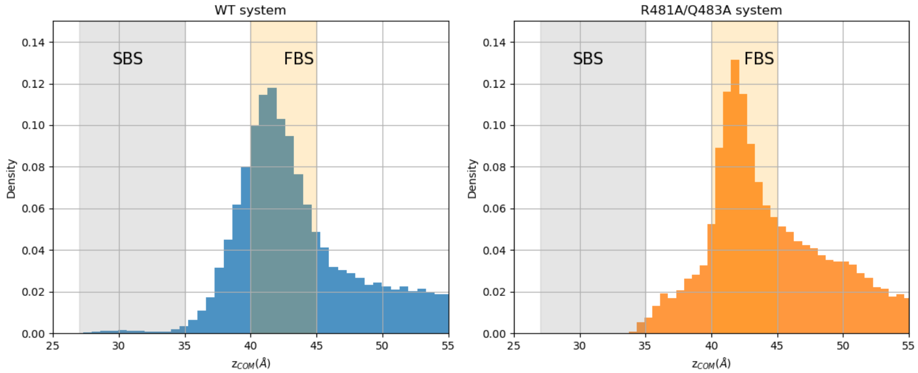

Figure S1: **Distribution of ligand positions along the membrane normal.** Distribution of the z-component of the center-of-mass (COM) position of the Fe^{3+}–enterobactin complex during metadynamics-to-dynamics simulations in the wild-type system (left) and in the double mutant (right). A non-negligible population at the secondary binding site (SBS) is observed only for the wild-type system.

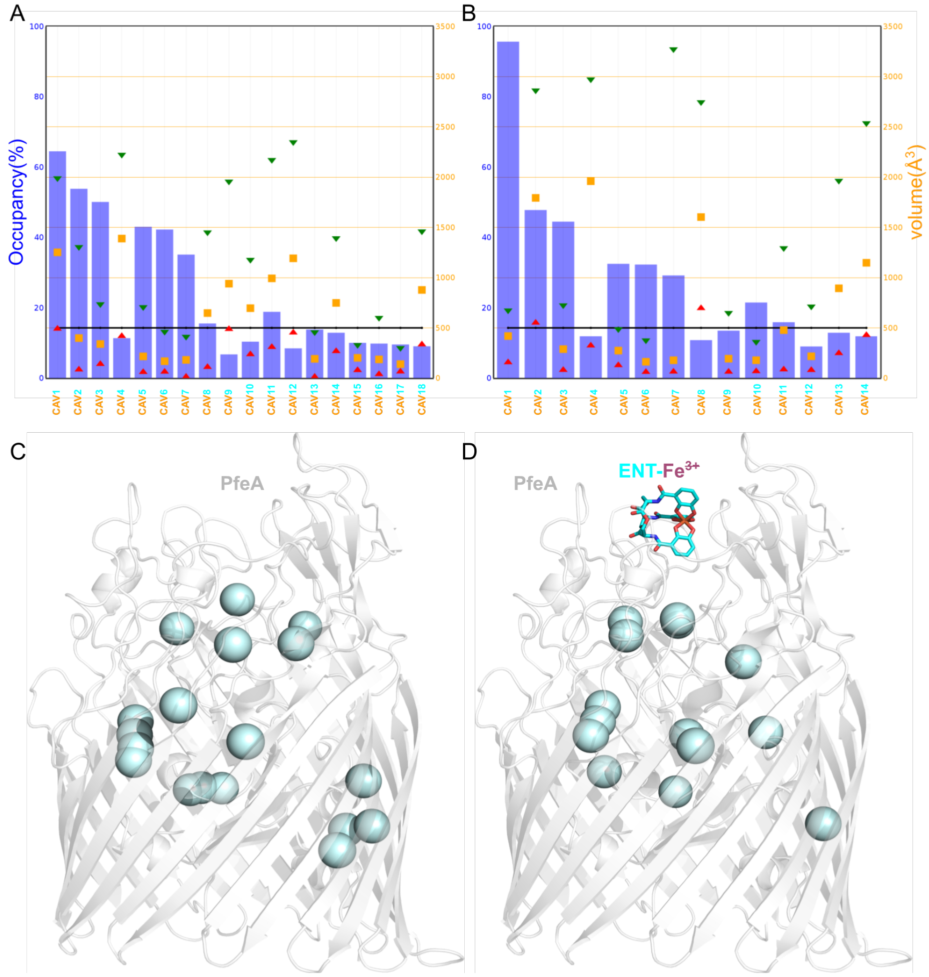

Figure S2: **Volume analysis of internal cavities in PfeA.** Volume analysis of the apo PfeA transporter (A, C) and PfeA bound to Fe^{3+}–enterobactin (B, D). The black horizontal line indicates a cavity volume of 500 Å^3. Orange, green, and red symbols represent the average, maximum, and minimum volumes, respectively, for each cavity population. In panels C and D, cavity volumes are represented as cyan spheres centered on the corresponding volume cluster centers.

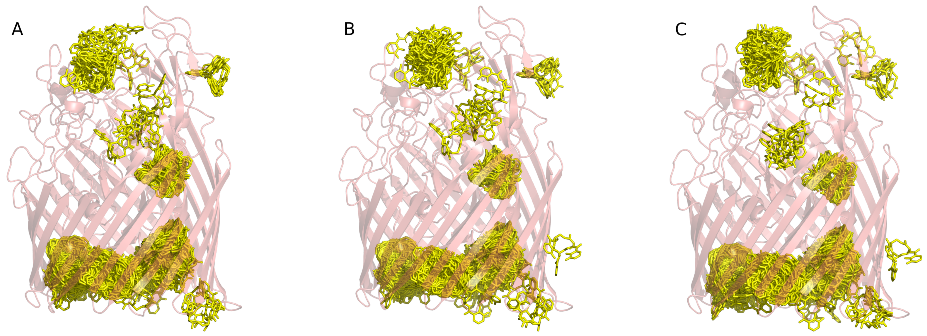

Figure S3: **Best docking poses identified.** The first, the second and the third best poses distributions over the docking campaing conducted on MD snapshots depicted in A, B, and C, respectively.

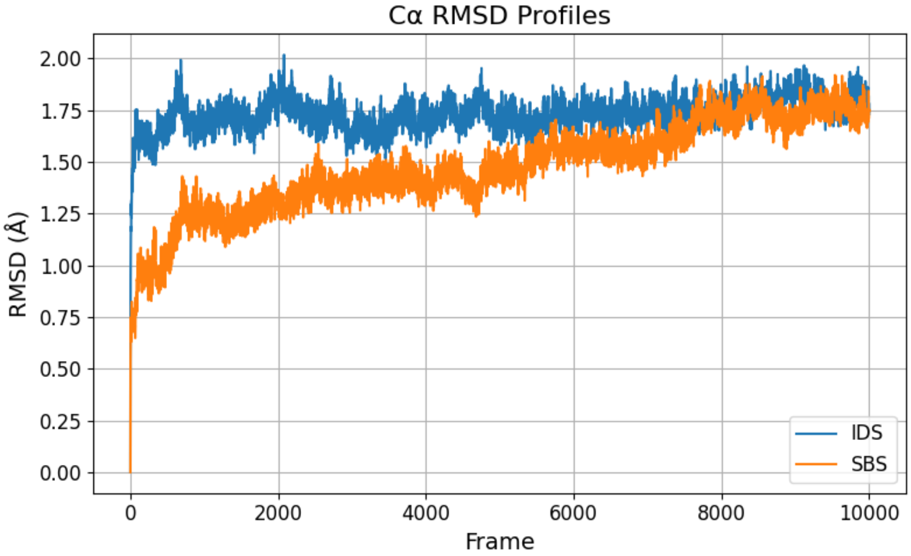

Figure S4: **Structural stability of PfeA during ligand binding.** Backbone RMSD profiles of PfeA when the ligand is localized at the internal docking site (IDS, blue) and at the secondary binding site (SBS, orange).

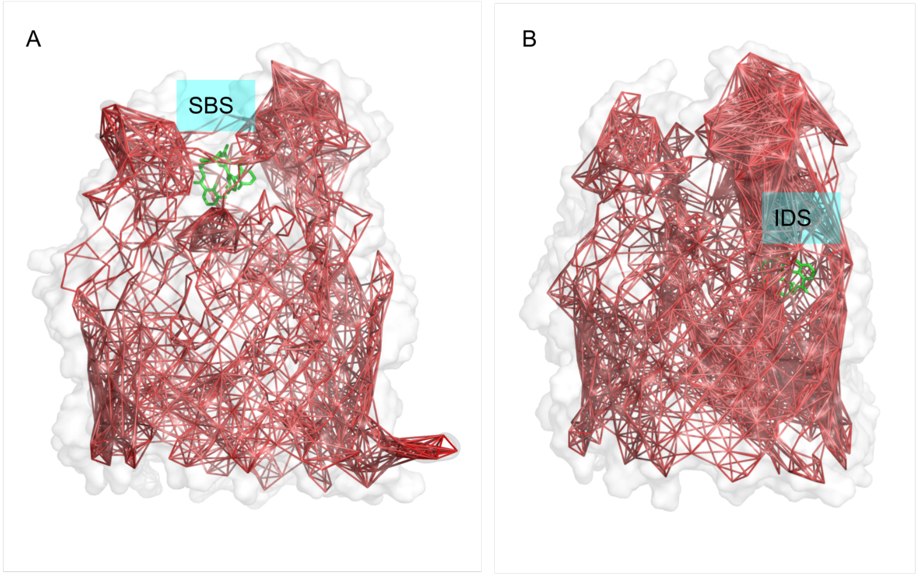

Figure S5: **Long-range correlation analysis of protein motions.** C_α–C_α cross-correlation matrices computed when the ligand is bound at the secondary binding site (panel A) and at the internal docking site (panel B).

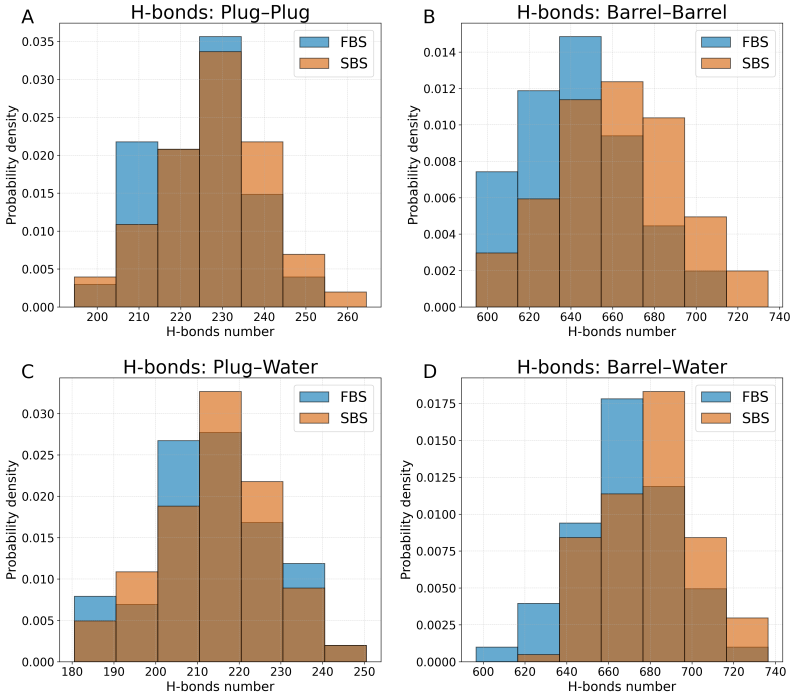

Figure S6: **Redistribution of hydrogen-bonding interactions upon ligand migration from the first to the secondary binding site.** (**A**) Distribution of hydrogen bonds between the plug domain and itself (plug–plug) when Fe^{3+}–enterobactin is bound at the first binding site (FBS, blue) and at the secondary binding site (SBS, orange). (**B**) Distribution of hydrogen bonds within the barrel domain (barrel–barrel) for FBS and SBS binding states. (**C**) Distribution of hydrogen bonds between the plug domain and surrounding water molecules (plug–water) for FBS and SBS. (**D**) Distribution of hydrogen bonds between the barrel domain and water molecules (barrel–water) for FBS and SBS. Ligand migration from the FBS to the SBS is accompanied by a redistribution of hydrogen-bonding interactions among protein domains and solvent, consistent with local reorganization at the plug–barrel interface.

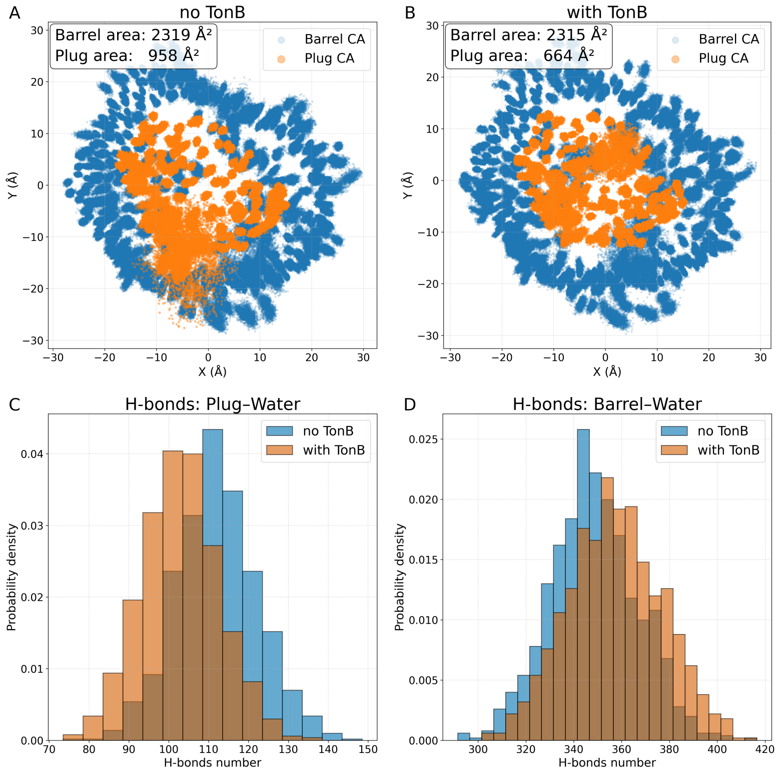

Figure S7: **TonB-induced reorganization of plug–barrel geometry and hydration.** (**A**) Projection of C_α atoms of the barrel (blue) and plug (orange) domains in the absence of TonB, with the corresponding convex-hull areas reported. (**B**) Same representation in the presence of TonB binding, showing a reduced projected area of the plug domain while the barrel area remains essentially unchanged. (**C**) Distribution of hydrogen bonds between the plug and surrounding water molecules with (orange) and without (blue) TonB bound. (**D**) Distribution of hydrogen bonds between the barrel and water molecules with (orange) and without (blue) TonB bound. TonB binding leads to a redistribution of hydration interactions, characterized by reduced plug–water hydrogen bonding and increased barrel–water interactions, consistent with a weakening of the plug–barrel interface.

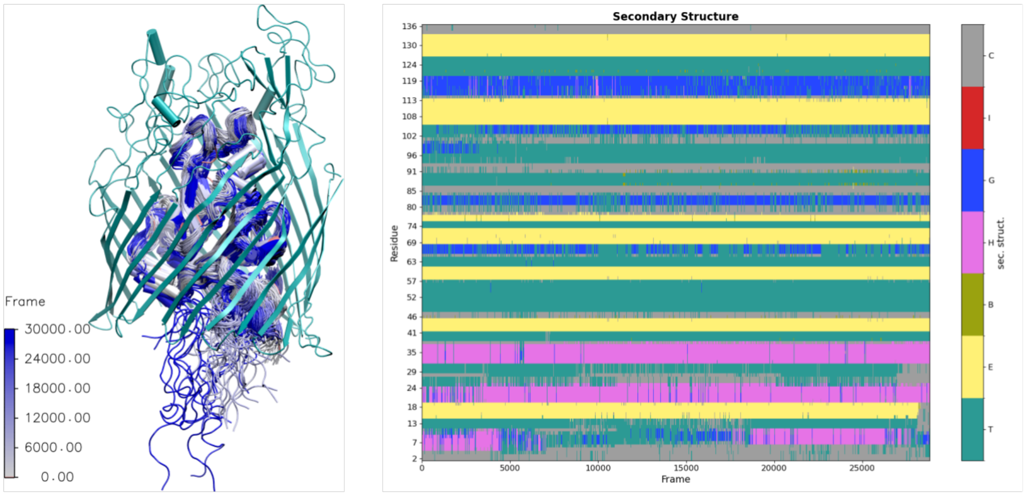

Figure S8: **Plug conformational dynamics under grid-biased metadynamics.** Analysis of conformations extracted from metadynamics simulations in which a grid bias was applied to the plug region. Left: superposition of representative conformers. Right: secondary structure analysis of the plug performed using the timeline tool in VMD.

Supporting Movie

Movie S1. Trajectory of Fe^{3+}–enterobactin metadynamics simulations with bias applied to plug–barrel interactions. The ligand diffuses from the first binding site (FBS) toward the periplasmic space through the secondary binding site (SBS) and the internal docking site (IDS).
